# Efficacy of Three Interdental Cleaning Methods for Peri-Implant Health Maintenance of Single Implant-Supported Crowns: A Randomised Clinical Trial

**DOI:** 10.3290/j.ohpd.b4854607

**Published:** 2024-01-15

**Authors:** Hani S. AlMoharib, Mansour H. AlAskar, Essam A. Abuthera, Khalid A. Alshalhoub, Faisal K. BinRokan, Nawaf S. AlQahtani, Hossam W. Almadhoon

**Affiliations:** a Assistant Professor, Periodontics and Community Dentistry, College of Dent, King Saud University, Riyadh, Saudi Arabia. Supervision, conceptualization, investigation, methodology, analysis, wrote and reviewed the manuscript.; b Professor, Periodontics and Community Dentistry, College of Dent, King Saud University, Riyadh, Saudi Arabia. Methodology, investigation, wrote the manuscript.; c Intern Dentist, College of Dentistry, King Saud University, Riyadh, Saudi Arabia. Investigation, data collection, wrote the manuscript.; d Intern Dentist, College of Dentistry, King Saud University, Riyadh, Saudi Arabia. Methodology, statistical analysis.; e Intern Dentist, College of Dentistry, King Saud University, Riyadh, Saudi Arabia. Methodology, statistical analysis.; f Dentist, Faculty of Dent, Al-Azhar University-Gaza, Gaza Strip, Palestine. Supervision, conceptualisation, investigation, methodology, wrote and reviewed the manuscript.

**Keywords:** dental implant, interdental aid, oral hygiene, single crowns, water floss

## Abstract

**Purpose:**

To compare the effectiveness of an interproximal brush, a water flosser, and dental floss in removing plaque and reducing inflammation around implant-supported crowns.

**Materials and Methods:**

A randomised controlled trial was conducted involving 45 participants with implant-supported single crowns. The participants were randomly assigned to three groups: interproximal brush, water flosser, and dental floss. Plaque index scores, gingival index scores, and interleukin-6 (IL-6) levels were assessed at baseline and after a two-week period. Statistical analysis was performed to compare the outcomes among the groups.

**Results:**

Following the second visit, improvements in plaque control were observed across all three interdental cleaning methods. The water flosser demonstrated a slight reduction in IL-6 levels (60.17 ± 3.07 vs 58.79 ± 4.04) compared to the initial visit, although this decrease was not statistically significant. Conversely, both the interdental brush and dental floss exhibited a slight increase in IL-6 levels at the second visit (60.73 ± 2.93 and 55.7 ± 10.64, respectively) compared to the mean at the first visit (58.38 ± 3.24 and 54.6 ± 2.22, respectively). Among the groups, only the interproximal brush demonstrated a statistically significant difference in IL-6 levels (p=0.008), while no statistically significant differences were observed in the dental floss and water flosser groups.

**Conclusion:**

Within the study’s limitations, our findings suggest that all three methods of interdental cleaning effectively improve plaque control and reduce gingival inflammation. However, using a water flosser appears to reduce inflammation more effectively, highlighting its potential advantage over the other two methods. Further research is needed to evaluate the long-term efficacy and impact of these methods on implant survival.

As the popularity of dental implants continues to grow, clinicians face challenges maintaining these complex restorations.^17^ Implant-supported crowns are a common treatment option for missing teeth. These crowns offer high clinical survival rates and long-term patient satisfaction.^6^ However, compared to tooth-supported prostheses, implant-supported crowns have a lower success rate due to their greater susceptibility to mechanical complications.^10^ While the main focus of implant dentistry has traditionally been on osseointegration, there is now a stronger emphasis on ensuring the long-term health of the peri-implant hard and soft tissues.^12,17^ This objective can be accomplished by employing a combination of suitable professional care, patient collaboration, and efficient home care.^17,21^

Consequently, it is essential to initiate personal oral hygiene practices at the time of an implant-supported crown placement, which should involve the utilisation of supplementary aids to ensure thorough cleaning of the restoration.^33^

Dental plaque, being the primary cause of periodontal diseases^9,20^ and a risk factor for peri-implantitis,^35^ poses a significant challenge as conventional toothbrushes are unable to adequately access the proximal surfaces, interproximal areas, and hard-to-reach regions surrounding fixed prostheses.^13^ To control plaque in these areas, additional methods such as dental floss and interdental brushes have been used, along with the use of water flossers to remove nonadherent bacteria and debris from the oral cavity.^27^ These adjunctive aids can effectively remove biofilm,^15^ reduce inflammation and pocket depth,^15,19^ and prevent the occurrence of peri-implant diseases that may lead to implant loss.^34^

Despite the availability of various interdental cleaning options, there is limited research on the most effective method for maintaining peri-implant health and preventing implant failure. This knowledge gap highlights the need for further investigation in this area. Therefore, this study aimed to evaluate the effects of an interproximal brush, a water flosser, and dental floss on plaque control, gingival health and the level of interleukin-6 (IL-6), a biomarker for peri-implant inflammation.^11^ The primary outcome measures were the changes in plaque index scores, gingival scores, and IL-6 levels before and after a two-week period of using the interdental cleaning methods. The test hypothesis was that these methods have varying effects on plaque control and the level of IL-6. This should provide a better understanding of the most effective methods of home care and optimise patient education regarding interdental cleaning techniques.

## Materials and Methods

### Ethical Approval

This study was conducted in April and May 2021 at the Dental University Hospital of King Saud University (KSU), Riyadh, Saudi Arabia. Ethical approval for this study was obtained from the Institutional Review Board of King Saud University Medical City, Riyadh, Saudi Arabia (Res Project No. E-21-5751). All participants signed an informed-consent form at the beginning of the study.

### Study Design

The research was conducted as a single-blinded, three-group, parallel randomised clinical trial to determine and compare the effectiveness of three home care devices for oral health maintenance – an interproximal brush, a water flosser, and dental floss – in improving implant and peri-implant health parameters and indices in patents with implant-supported single crowns. The study participants were assigned to one of three groups through a computer-generated randomisation process. Randomisation was carried out at both the individual subject and implant levels, resulting in 15 implants being assigned to each group. The allocation ratio was 1:1:1, ensuring equal representation of each group. The study incorporated two main phases: an initial four-week recruitment stage, which was then followed by an evaluation stage where assessments took place on both the first and last days of a two-week period.

### Patient Population

In this study, strict inclusion and exclusion criteria were used to ensure that only individuals who met specific requirements were selected to participate. To be eligible for the study, participants had to be healthy, non-smokers, at least 18 years of age, and provide written consent to participate. Additionally, they were required to have a single implant-supported crown in the mandibular posterior region with a probing depth ≤ 5 mm, no radiographic bone loss, and a good quality restoration that was not inherently plaque retentive, which had been in place for four years between 2018 and 2021. Certain individuals were excluded from the study based on specific conditions, such as being immunocompromised or taking steroids, being pregnant or intending to become pregnant during the study, having taken antibiotics within the past three months, or having received periodontal treatment within the past month.

### Procedure

Forty-five subjects were randomly allocated to one of the study groups: interproximal brush (group A), water flosser (group B), or dental floss (group C), with fifteen participants in each group. Subjects attended two visits: the first visit comprised the baseline for evaluation, and the second visit was the end-point assessment. For the purpose of blinding, the naming of groups was not disclosed to the evaluator for the whole duration of the study to minimise bias.

- Group A: Subjects in this group were instructed to brush twice daily with a manual toothbrush for two minutes along with an interproximal brush (Oral-B Proxy brush, Procter & Gamble; Cincinnati, OH, USA).
- Group B: Subjects in this group were instructed to brush twice daily with a manual toothbrush for two minutes along with a cordless water flosser (H2Ofloss, Shenzhen BFT Electrical Appliances Manufacturing; Shenzhen, China).
- Group C: Subjects in this group were instructed to brush twice daily with a manual toothbrush for two minutes along with dental floss (Oral-B Glide Floss). Subjects in all groups were instructed to use fluoridated toothpaste.

The primary outcome measures collected in this study included the simplified plaque index, gingival index by Löe and Silness, and IL-6 as an inflammatory parameter, measured in duplicate using enzyme-linked immunosorbent assays (ELISA). A human interleukin-6 (IL-6) (Quantikine ELISA Kit; R&D Systems; Minneapolis, MN, USA) kit was used on the peri-implant crevicular fluid (PICF), according to the manufacturer’s instructions.

All subjects were evaluated at two sequential appointments. At the end of the first visit, the subject was introduced to the allocated oral hygiene aid, its use was demonstrated and verbal as well as written instructions were given. The subject presented at the 2nd visit in 2 weeks, where the same data were collected again for comparison with baseline. One blinded examiner collected the entire sample during both visits and kept separate records for each previous and current examination.

### Data Analysis

Non-parametric tests were conducted using SPSS version 22, along with the Kruskal-Wallis test between factors at α= 0.05 and power = 1.00. Descriptive statistics were used to present the output of the analysed data. In addition, the Wilcoxon signed-ranks test was done to determine the difference in mean values between the two visits. A p-value < 0.05 was considered statistically significant at a 95% confidence interval. To ensure a reliable and statistically significant difference, the study determined a sample size of 15 implants per group, with an anticipated average effect size of 0.9 and a desired power of 80%. The sample size was determined based on a previous study that examined the cleaning efficacy of two different interdental brushes.^28^

## Results

**Fig 1:**
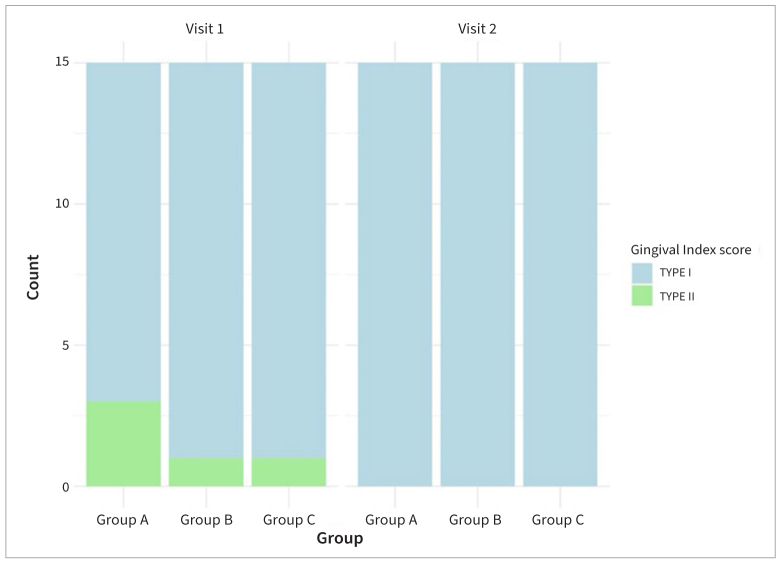
Stacked bar plot of gingival index by group and visit.

The study included participants with 45 implants. All of them had similar baseline characteristics, with a mean probing depth of 3 mm ( ± 1). The majority of participants had a plaque index score ranging between 20% and 40% and a type I gingival index. At the second visit, improvements were observed in the gingival index for most participants (Fig 1).

Table 1 displays the mean IL-6 levels for each group at the first and second visits. At the initial visit, the overall IL-6 levels of the subjects were comparable to each other, with an average of 57.79. Similarly, at the second visit, the mean IL-6 level was 58.47. The water-flosser group showed a non-significant decrease in the mean IL-6 level at the second compared to the first visit (60.17 ± 3.07 vs 58.8 ± 4.04, respectively). In contrast, the interdental-brush and dental-floss groups showed a non-significant increase in IL-6 levels at the second visit (60.73 ± 2.93 and 55.7 ± 10.64, respectively) compared to the mean at the first visit (58.38 ± 3.24 and 54.6 ± 2.22, respectively). Among the three groups, only the water-flosser group exhibited a decrease in IL-6 levels after using the interdental aid. However, a statistically significant difference was observed only within group A, the interproximal brush (p = 0.008), while no statistically significant differences were found in the interdental-brush and dental-floss groups.

**Table 1:** Presents the mean IL-6 parameter levels for the implants PICF taken on the 1st and 2nd visits and the differences in these levels among the three groups

Group	Follow-up	N	Mean	Std. Deviation	p-value
A (Interdental brush)	Baseline	15	58.385	3.238	0.008*
	Two weeks	15	60.735	2.934	
B (water flosser)	Baseline	15	60.172	3.067	0.207
	Two weeks	15	58.797	4.015	
C (Dental floss)	Baseline	15	54.604	12.221	0.760
	Two weeks	15	55.701	10.649	

N: number; *statistically significant.

## Discussion

The maintenance of peri-implant health in patients with implant-supported single crowns is of paramount importance to ensure long-term implant success. While various interdental cleaning methods are available, studies which specifically investigated the efficacy of these methods in maintaining peri-implant health are scarce.^23,26^ In this study, we aimed to comparatively assess three commonly used interdental cleaning methods: an interproximal brush, a water flosser, and dental floss.

In our study, all three interdental cleaning methods yielded notable improvements in plaque control, as demonstrated by reduced plaque index scores. These findings are consistent with previous studies which highlighted the effectiveness of interdental cleaning devices in removing plaque and maintaining oral hygiene.^7,31,36^ The reduction in plaque accumulation is crucial for preventing peri-implant diseases such as peri-implant mucositis and peri-implantitis, which are strongly associated with increased plaque levels.^28^ Furthermore, our study observed a decrease in bleeding on probing for most participants, indicating an improvement in gingival health with all three interdental cleaning methods. Similarly, a systematic review by Worthington et al^36^ suggested the beneficial effects of interdental cleaning devices in reducing bleeding and gingival indices. The observed improvement in both plaque and gingival index scores in addition to decreased bleeding on probing across all groups suggest that implementing a regular interdental cleaning regimen is crucial for maintaining peri-implant health. These improvements align with the literature which shows that appropriate home care devices are essential for reducing bacterial plaque accumulation and subsequent inflammation around dental implants.^36^

In the literature, many studies have compared the effectiveness of interdental brushes with dental floss or a water flosser in different populations and periodontal conditions. For instance, the split-mouth randomised clinical trial by Imai et al^18^ assessed the effectiveness of an interdental brush and dental floss in 30 volunteers with a minimum of 4 bleeding sites per side for 12 weeks. They reported no difference between either method for plaque removal. A similar study by Noorlin et al,^19^ conducted on ten patients with periodontal disease, reported that both devices exert similar beneficial effects on plaque scores. A systematic review conducted by Sälzer et al^31^ found that the majority of the existing studies do not provide substantial evidence supporting the overall effectiveness of flossing compared to other interdental plaque control. However, A Cochrane review published in 2019^36^ found that most of the included studies which compared the effectiveness of interdental brushes with dental floss or water flossers demonstrated that water flossers might be more effective than dental floss or interdental brushes in reducing bleeding.^36^

In the context of implant maintenance, there is a notable lack of research examining the comparative effectiveness of different interproximal cleaning methods.^22^ However, in recent years, a growing number of studies have been conducted to assess interproximal cleaning methods and determine which is the most effective. A cross-over clinical trial by Luz et al^23^ that included 12 implants and was followed-up for two months found that interdental brushes are more effective than dental floss in removing proximal biofilm around implants. Another study, carried out by Magnuson et al^26^ investigated the effect of water flossing on the reduction of bleeding on probing among patients with implant-supported prostheses. They found that the water flosser statistically significantly reduced the bleeding on probing after 30 days of use compared to dental floss. Contrary to these findings, our study revealed that the water flosser did not statistically significantly affect the hygiene of implant-supported crowns, as mean plaque scores among patients on both visits were nearly similar. In addition, the effect of a water flosser was compared with two other interdental cleaning methods, including interdental brushes and dental floss. However, no statistically significant differences were observed.

IL-6 levels were measured in our study to further evaluate the impact of the interdental cleaning methods on inflammation. IL-6 is a pro-inflammatory cytokine that plays a crucial role in periodontal destruction.^32^ We assessed IL-6 levels as a potential marker for evaluating the inflammatory status around the implants.^11^ Interestingly, we observed varying trends in IL-6 levels among the different interdental cleaning method groups. The overall IL-6 levels at both visits were comparable among the subjects, indicating a similar inflammatory status at baseline. Among the three investigated methods, the water flosser demonstrated a noticeable reduction in IL-6 levels, a key marker for inflammation in implant-related issues such as peri-implantitis.^26^ This finding suggests an advantage in reducing inflammation compared to the other groups. Several studies have reported the anti-inflammatory effects of water flossers, as they effectively eliminate loosely attached debris and bacteria from interdental areas, thereby reducing the inflammatory response.^14,16,25,30^ Additionally, the use of water flossers has been proven to diminish inflammation in individuals with localised mild to moderate periodontitis and diabetes by reducing the levels of pro-inflammatory cytokines (such as IL-1β and PGE2) in the gingival crevicular fluid.^1,8^ However, the absence of studies evaluating IL-6 levels as an inflammatory parameter in the context of dental implants limits the contextualisation of our findings within existing research.

In contrast, although interdental-brush and dental-floss use were associated with improvements in plaque index scores and bleeding on probing, no statistically significant reductions in IL-6 levels were found. These results imply the limitations of both cleaning methods in addressing inflammation around dental implants, which might suggest that the flushing action provided by the water flosser has a superior effect on the removal of bacterial plaque and the subsequent inflammatory process.^27^ However, it is also possible that the mechanical action of interproximal brushes and dental floss in removing plaque and biofilms may have stimulated a local inflammatory response. Additionally, individual variations in oral hygiene practices and technique proficiency could have influenced these results.^4^ However, the differences in IL-6 levels observed among both groups were not statistically significant. These findings may be attributed to the relatively small sample size and the short duration of the study.

Our study findings support the clinical preference for interproximal brushes or water flossers over dental floss for maintaining peri-implant health. This preference is justified by the potential risks associated with flossing, such as exposing rough implant surfaces and causing fraying. Therefore, using interproximal brushes or water flossers presents a more favourable alternative.^34^ This is further supported by Ng and Lim,^27^ who recommend interdental brushes and water flossers over dental floss for cleaning around dental implants. Several hypotheses have been presented to describe this effect, with supra-gingival irrigation changing the microbial composition and reducing the virulence of dental plaque.^37^ Despite initial reservations among some clinicians, scientific evidence indicates that using a pulsating oral-irrigation device, such as a water flosser, is both safe and beneficial.^31^ Extensive use by the general public over the years has not shown any adverse effects, and the device effectively removes bacteria without pushing them into the periodontal pockets.^24^

Despite the valuable insights provided by our study into the efficacy of interdental cleaning methods in managing peri-implant health, several limitations should be acknowledged. First, the sample size was relatively small, which may have limited the detection of smaller differences in IL-6 levels between the groups. Future studies with larger sample sizes are needed to validate and strengthen the results. Second, the study duration (2 weeks) was relatively short, and the evaluation period may not have been sufficient to capture long-term effects of the interdental cleaning methods on peri-implant health. Longer follow-up periods would provide a more comprehensive understanding of the sustained efficacy of these methods. Third, the study relied on self-reported oral hygiene practices, which may introduce recall bias or subjective interpretation of adherence. Future research could benefit from objective measures, such as additional inflammatory biomarkers (i.e., TNFα, MMP-8), to provide more accurate and reliable data.^2,3^ Lastly, future research should also consider evaluating patient-reported outcomes, such as satisfaction, comfort, and ease of use to assess the acceptability of and compliance with different interdental cleaning methods among patients.

## Conclusions

Within the study’s limitations, our findings indicate that all three interdental cleaning methods (interproximal brush, water flosser, and dental floss) effectively improve plaque control and reduce gingival inflammation. While the impact on inflammation around implants as assessed by IL-6 levels varied across the methods, the trend towards decreased inflammation with the water flosser highlights its potential advantage. Dental professionals should continue emphasising the importance of regular oral hygiene practices and interdental cleaning in ensuring long-term implant success. Further studies are needed to evaluate the long-term efficacy of these interdental cleaning methods and their impact on implant survival.
